# Radial probe endobronchial ultrasound using a guide sheath for peripheral lung lesions in beginners

**DOI:** 10.1186/s12890-018-0704-7

**Published:** 2018-08-13

**Authors:** Jung Seop Eom, Jeong Ha Mok, Insu Kim, Min Ki Lee, Geewon Lee, Hyemi Park, Ji Won Lee, Yeon Joo Jeong, Won-Young Kim, Eun Jung Jo, Mi Hyun Kim, Kwangha Lee, Ki Uk Kim, Hye-Kyung Park

**Affiliations:** 1 Department of Internal Medicine, Pusan National University School of Medicine, 179 Gudeok-ro, Seo-gu, Busan, 602-739 South Korea; 2 Department of Radiology, Pusan National University School of Medicine, Busan, South Korea; 3 Biostatistics Team of Regional Center for Respiratory Diseases, Pusan National University School of Medicine, Busan, South Korea; 4 Biomedical Research Institute, Pusan National University Hospital, Busan, South Korea

**Keywords:** Bronchoscopy, Ultrasound, Complication, Diagnosis, Lung neoplasms

## Abstract

**Background:**

The diagnostic yields and safety profiles of transbronchial lung biopsy have not been evaluated in inexperienced physicians using the combined modality of radial probe endobronchial ultrasound and a guide sheath (EBUS-GS). This study assessed the utility and safety of EBUS-GS during the learning phase by referring to a database of performed EBUS-GS procedures.

**Methods:**

From December 2015 to January 2017, all of the consecutive patients who underwent EBUS-GS were registered. During the study period, two physicians with no previous experience performed the procedure. To assess the diagnostic yields, learning curve, and safety profile of EBUS-GS performed by these inexperienced physicians, the first 100 consecutive EBUS-GS procedures were included in the evaluation.

**Results:**

The overall diagnostic yield of EBUS-GS performed by two physicans in 200 patients with a peripheral lung lesion was 73.0%. Learning curve analyses showed that the diagnostic yields were stable, even when the procedure was performed by beginners. Complications related to EBUS-GS occurred in three patients (1.5%): pneumothorax developed in two patients (1%) and resolved spontaneously without chest tube drainage; another patient (0.5%) developed a pulmonary infection after EBUS-GS. There were no cases of pneumothorax requiring chest tube drainage, severe hemorrhage, respiratory failure, premature termination of the procedure, or procedure-related mortality.

**Conclusions:**

EBUS-GS is a safe and stable procedure with an acceptable diagnostic yield, even when performed by physicians with no previous experience.

**Electronic supplementary material:**

The online version of this article (10.1186/s12890-018-0704-7) contains supplementary material, which is available to authorized users.

## Background

Until now, the pathological diagnosis of a peripheral lung lesion was usually made by transthoracic needle biopsy, surgical resection, or bronchoscopy; however, transbronchial lung biopsy using conventional bronchoscopy has a low diagnostic yield [1]. Technological advances have developed peripheral bronchoscopy as a useful and minimally invasive procedure [2–4]. Moreover, the diagnostic yield of peripheral bronchoscopy has been greatly improved by a combined modality consisting of radial probe endobronchial ultrasound and a guide sheath (EBUS-GS) [5].

Based on the results of previous studies, EBUS-GS for peripheral lung lesions is considered a relatively safe procedure with an acceptable diagnostic yield [6, 7]. Given its widespread use, complications might be expected, particularly when the procedure is performed by inexperienced physicians. Previous meta-analyses determined an overall complication rate between 0 and 7.4%, but zero mortality [6, 7]. In a recent large-scale study of 965 patients, the rates of iatrogenic pneumothorax, pneumothorax requiring chest tube drainage, and pulmonary infection was 0.8%, 0.3%, and 0.5%, respectively, which were markedly lower than the rate related to transthoracic needle biopsy [1, 8, 9]. Breakage of the radial probe during EBUS occurred in 0.4% of the patients. However, there are no clinical data regarding the diagnostic yields, learning curve, and safety profile for procedures performed by inexperienced physicians. Thus, using a prospectively collected database, we determined the learning curve and safety profile of EBUS-GS when performed by beginners. We also analyzed the durability of the radial probe and GS in those procedures.

## Methods

### Study population

From December 2015 to January 2017, a retrospective study was conducted to investigate the clinical outcomes of patients undergoing EBUS-GS performed by beginners. During the study period, two physicians, neither of whom had previously performed EBUS-GS or radial probe EBUS only, began EBUS-GS at Pusan National University Hospital, a university-affiliated, tertiary referral hospital in Busan, South Korea. Before starting EBUS-GS, the two beginners both had 4 years of experience with conventional bronchoscopy and 3 years of experience with convex probe EBUS (700 conventional bronchoscopies and 200 convex probe EBUS per year by each physician). All of the consecutive patients with a peripheral lung lesion, who underwent EBUS-GS performed by one of the physicians, were prospectively registered. For each physician, the first 100 consecutive patients who received EBUS-GS were included in the analyses. Prior to EBUS-GS, written informed consent was obtained from all of the patients. The Institutional Review Board of Pusan National University Hospital approved this study (No. E-2016084) and informed consent was waived due to the retrospective nature of this study and the anonymized personal information prior to analysis.

### Computed tomography and peripheral lung lesions

All of the chest computed tomography (CT) scans were performed within 2 weeks prior to EBUS-GS. The imaging parameters were 120 kVp and 100–250 mAs. The stored CT raw data were used to reconstruct images at a slice thickness of 0.625 mm and intervals of 0.625 mm. The size of each peripheral lung lesion was measured from the CT images, based on the mean diameter of the lesion on the axial lung window setting. A peripheral lung lesion was diagnosed when the location of the lesion was beyond the segmental bronchus [10]. The lesion was classified as ground-glass opacity, part-solid, or solid according to a visual assessment method based on CT attenuation and modified from a previous study [11].

### EBUS-GS and associated complications

All of the EBUS-GS procedures were performed during in-patient hospital stays. Before the procedure, a 20 MHz radial probe EBUS (UM-S20–17S; Olympus, Tokyo, Japan) and GS kit (K-201; Olympus, Tokyo, Japan) were prepared according to the standard method of Kurimoto [5]. Patients under conscious sedation with intravenous midazolam and fentanyl underwent conventional bronchoscopy with a 4.0 mm flexible bronchoscope (BF-P260F; Olympus, Tokyo, Japan) to inspect the large airway. Lidocaine (2%) was applied to the tracheobronchial tree via the working channel of the bronchoscope. Following conventional bronchoscopy, the bronchoscope was advanced into the bronchus of interest as far as possible under direct vision based on the CT image. Thereafter, the GS-covered radial probe EBUS was advanced through the working channel of the bronchoscope until resistance was met. Then the probe was pulled back slightly to allow ultrasound scanning under X-ray fluoroscopic guidance. When the location of the target lesion was identified using EBUS, the probe was removed while the GS was kept in place for subsequent brush cytology and forceps biopsy. According to the sonographic features of the target lesion, the relationship between the lung lesion and GS was classified into three patterns, as previously reported [2, 5, 12]: within, adjacent to, and outside the lesion (Additional file 1). Brush cytology and a forceps biopsy via the GS were performed under X-ray fluoroscopy for the histological examination. Endobronchial ultrasound guided transbronchial needle aspiration was not simultaneously performed for mediastinal lymph node sampling during EBUS-GS. All of the procedures were performed without the assistance of virtual bronchoscopy navigation or an electromagnetic navigation system [13, 14]. If the lesion was located outside the EBUS probe, the sampling approach, whether brush cytology, forceps biopsy, or bronchial washing, was selected at the discretion of the bronchoscopist. A representative case of EBUS-GS for a peripheral lung lesion is shown in Additional file 2. To determine if iatrogenic pneumothorax had developed, initial chest radiographs were obtained 4 h after the procedure, and follow-up chest X-rays the following day. Severe hemorrhage was defined as endobronchial bleeding requiring transfusion, intubation, or an interventional procedure. Respiratory failure requiring intubation, pulmonary infection, air embolism, or premature termination of the procedure due to another unexpected complication was also recorded. X-ray fluoroscopy was performed to detect whether the GS had broken during the procedure. To identify breakage of the radial probe EBUS, an ultrasound image of the withdrawn probe held in the air was taken after the procedure, and saved on a picture archiving and communication system (Additional file 2).

### Statistical analysis

Statistical analyses were performed using SPSS version 22.0 (SPSS Inc., Chicago, IL, USA). The results are presented as numbers (percentages) or medians (interquartile ranges [IQRs]), as appropriate. Pearson’s chi-square test or Fisher’s exact test was used for categorical variables and the Mann–Whitney U-test was used for continuous variables. A *P*-value < 0.05 was considered statistically significant. To assess the learning curve of the procedure, cumulative sum (CUSUM) analyses were used to produce a learning curve for each physician. The definition of CUSUM analysis applied in this study was that of Bolsin and Colson (Additional file 3) [15]. A detailed description of the CUSUM analysis in this study is provided in Additional file 4.

## Results

### Study population

Two hundred patients with peripheral lung lesions were included in the study (100 patients per physician). Their baseline characteristics are shown in Table 1. The median mean lesion diameter was 26 mm (IQR, 20–37 mm). Using the radial probe EBUS, 162 (81.0%) of the lesions were identified as being ‘within’ image and 24 (12.0%) ‘adjacent to’ image. However, 14 lung lesions (7.0%) were invisible. According to the appearance of the peripheral lung lesions on CT, there were 170 solid (85.0%), 26 part-solid (13.0%), and 4 ground-glass opacity (2.0%) lesions. The median number of brush cytology tests and forceps biopsies, performed via the GS, was 3 (IQR, 3–3) and 6 (IQR, 6–7), respectively. The overall EBUS-GS time was 20 min (IQR, 14–25 min). In addition, no significant difference in baseline characteristics was observed between the 100 study patients in which EBUS-GS was performed by one of the physicians (Additional file 5).

**Table 1:** Baseline characteristics of 200 study patients

Variables	Median (IQR) or No. (%)
Age, years	67 (59–73)
Male gender	129 (64.5)
Mean diameter of lesion, mm	26 (20–37)
Character of lesion on computed tomography	
Solid	170 (85.0)
Part-solid	26 (13.0)
Ground-glass opacity	4 (2.0)
Location of the lesion	
Right upper lobe	54 (27.0)
Right middle lobe	12 (6.0)
Right lower lobe	48 (24.0)
Left upper division	45 (22.5)
Left lingular division	6 (3.0)
Left lower lobe	35 (17.5)
Endobronchial ultrasound image	
Within	162 (81.0)
Adjacent to	24 (12.0)
Outside	14 (7.0)
The number of brushing cytology tests performed via GS	3 (3–3)
The number of forceps biopsies performed via GS	6 (6–7)
Overall procedure time, min	20 (14–25)

*IQR* interquartile range, *GS* guide sheath

### Diagnostic yields

Table 2 lists the clinical diagnoses of the study patients. The overall diagnostic yield of EBUS-GS was 73.0%. Histological and cytological diagnoses were established in 146 (73.0%) and 42 (21.0%) of the 200 peripheral lung lesions, respectively. Diagnostic yields were significantly different among patients whose lesions had a mean diameter <  20 mm, 20–30 mm, and >  30 mm (46.8% vs. 80.8% vs. 81.3%, respectively, *P* < 0.001) (Table 3). No significant difference was observed in the diagnostic yield between solid and mixed lesions (75% vs. 69%, *P* = 0.553). However, the diagnostic yield of ground-glass opacity nodules was only 25%. Diagnostic yield “within the lesion” on EBUS findings was significantly higher than that of “adjacent and outside the lesion” on EBUS (80% vs. 58% vs. 14%, respectively, P < 0.001). In addition, the diagnostic yield obtained by the two physicians did not differ significantly (74.0% vs. 72.0%, *P* = 0.750).

**Table 2:** Clinical diagnosis of 200 patients who underwent EBUS-GS

Variables	No. (%)
Diagnosed with EBUS-GS (*n* = 146)	
Malignant disease	
Lung cancer	130 (89.0)
Colon cancer	2 (1.4)
Uterine cancer	1 (0.7)
Thyroid cancer	1 (0.7)
Hepatocellular carcinoma	1 (0.7)
Perivascular epithelioid cell tumor	1 (0.7)
Benign disease	
Pulmonary tuberculosis	5 (3.4)
Organizing pneumonia	4 (2.8)
Cryptococcosis	1 (0.7)
Undiagnosed with EBUS-GS (*n* = 54)	
Malignant disease	
Lung cancer	17 (31.5)
Mesothelioma	1 (1.9)
Breast cancer	1 (1.9)
Benign disease	
Chondroid hamartoma	1 (1.9)
Pulmonary tuberculosis	2 (3.7)
Non-tuberculous mycobacterial lung disease	1 (1.9)
Organizing pneumonia	2 (3.7)
IgG4-related disease	1 (1.9)
Unknown	28 (51.9)

EBUS-GS, transbronchial lung biopsy using radial probe endobronchial ultrasound and guide sheath; IgG4, immunoglobulin G4

**Table 3:** Diagnostic yield by EBUS-GS according to lesion size

Mean diameter, mm	No./Total (%)
< 20	22/47 (46.8)
20–30	59/73 (80.8)
> 30	65/80 (81.3)
Total	146/200 (73.0)

Diagnostic yields were significantly different among patients with lesions < 20 mm, 20–30 mm, and > 30 mm in mean diameter (*P* < 0.001)

### Identification of the learning curve

**Fig. 1:**
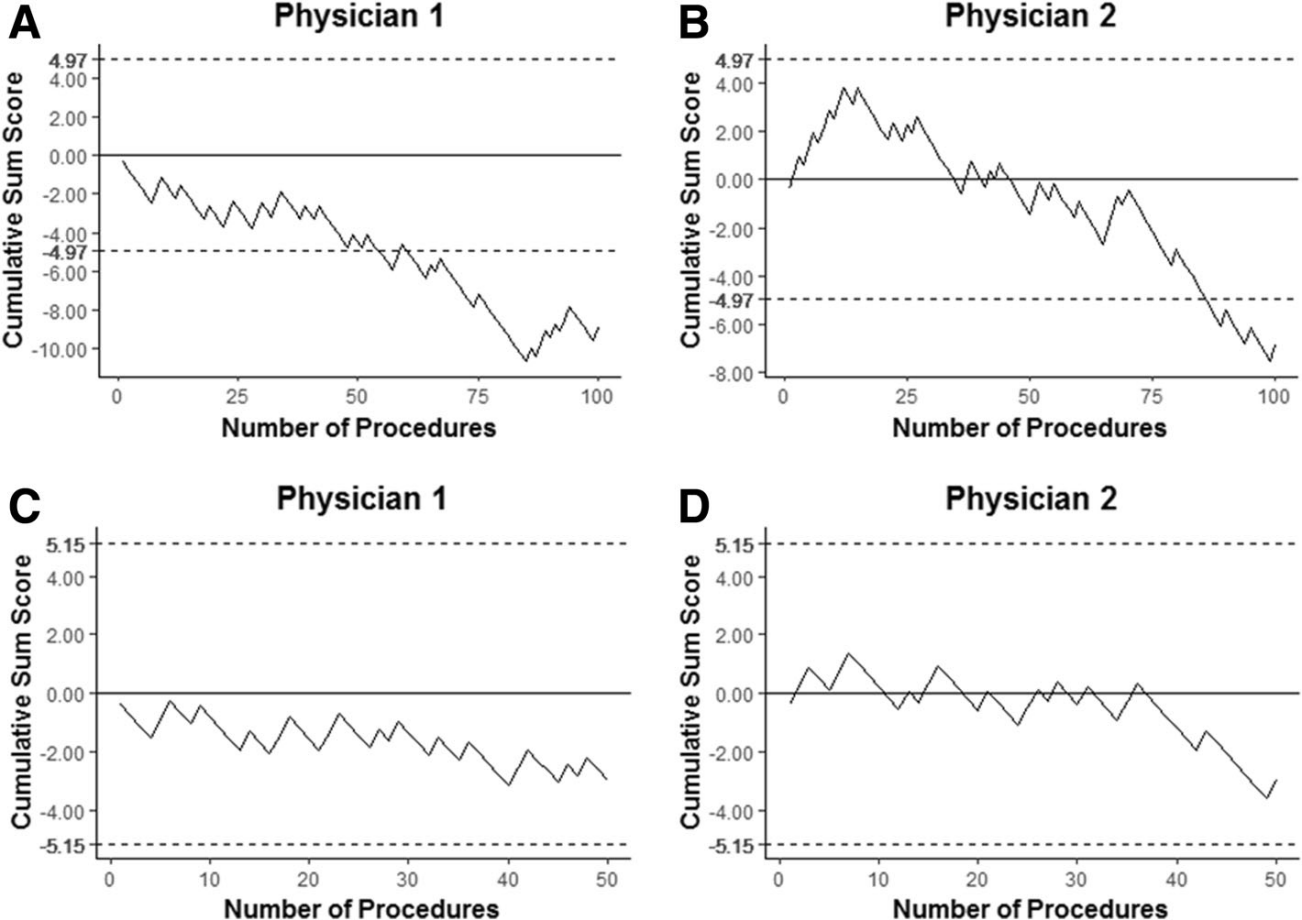
Cumulative sum analysis curves for the two physicians. (**a, b**) Analyses of the 100 patients evaluated by each physician. (**c**, **d**) Analyses of the consecutive 50 patients with lung lesions < 30 mm who underwent EBUS-GS by one of the two physicians

The results of the CUSUM analysis are presented as learning curves, in which a positive deflection represents false results and a negative deflection represents true results (Fig. 1). The curves show that the two physicians attained competence immediately and the curves remained below the predetermined decision interval throughout the study period (H1 = 4.97). In addition, the graphs of the two physicians crossed the lower decision boundary during the study period.

Additional CUSUM analyses were performed for 50 consecutive patients with peripheral lung lesions < 30 mm. The respective curves remained between the predetermined decision interval (H0 = − 5.15 and H1 = 5.15), again indicating that the physicians attained competence immediately, even when performing procedures involving small lung lesions.

### Complications

**Fig. 2:**
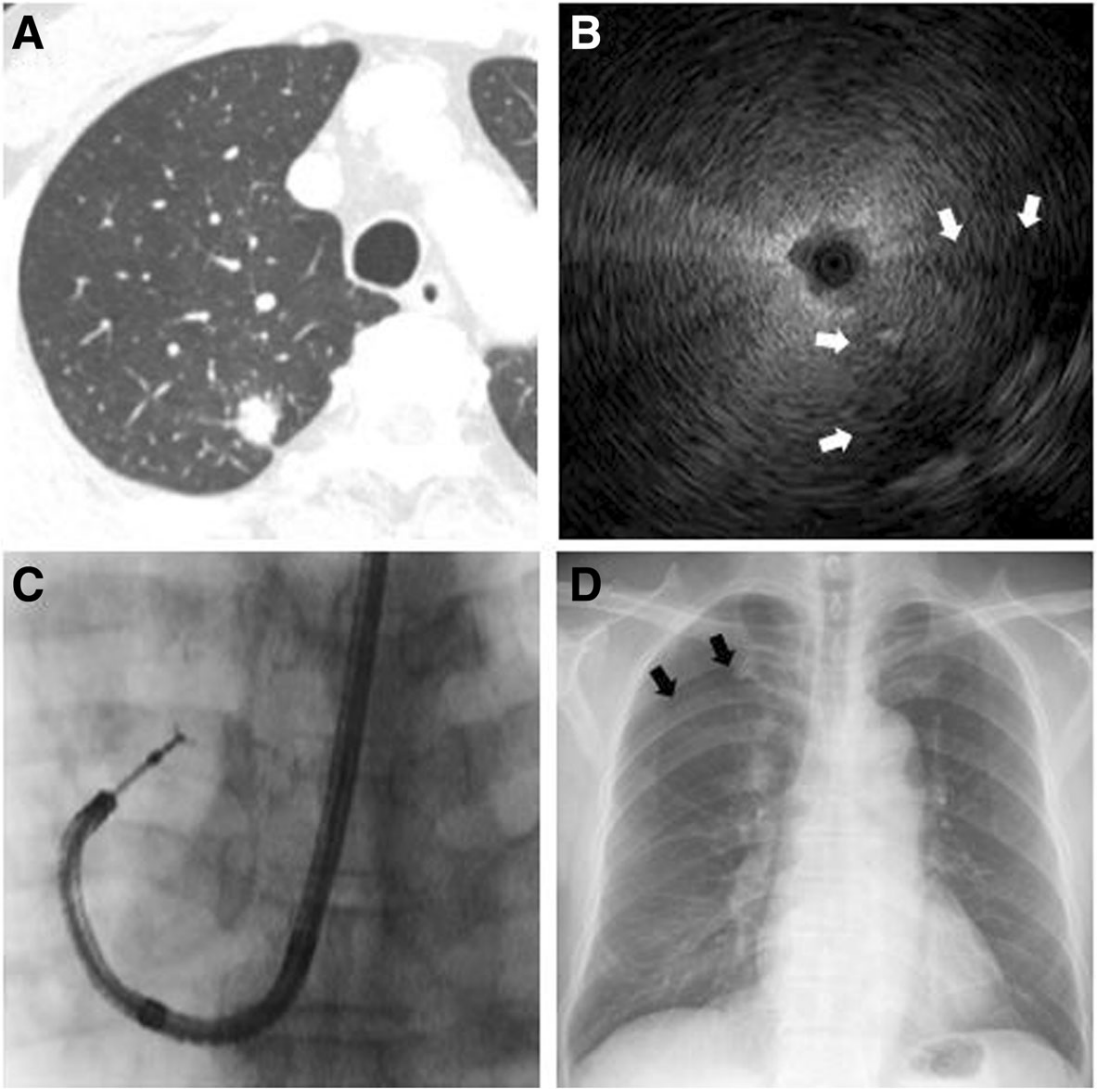
A patient who developed pneumothorax after the procedure.**a** A patient was admitted with a peripheral lung nodule measuring 15.1 mm at its greatest diameter and located in the right upper lobe, as seen on a chest computed tomography scan. **b** A radial probe endobronchial ultrasound (EBUS) image showed a hypoechoic area (white arrow) distinguishable from the normal aerated lung. **c** Under fluoroscopic guidance, transbronchial lung biopsy and brush cytology were performed via the guide sheath (GS). The diagnosis was adenocarcinoma. **d** Iatrogenic pneumothorax (black arrow) was identified on chest radiographs taken 4 h after EBUS-GS

**Fig. 3:**
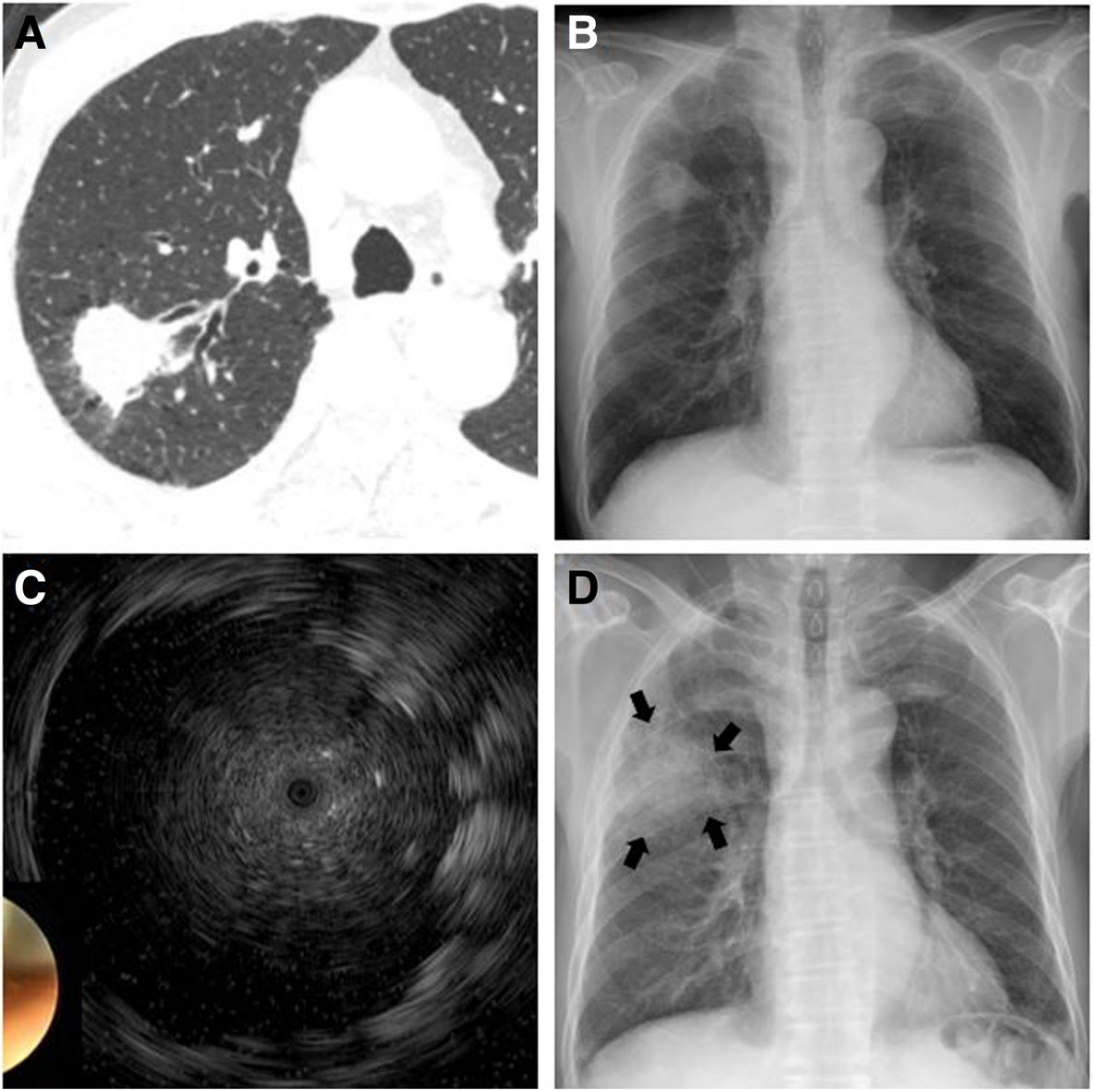
A patient who developed pneumonia after the procedure. **a** and **b** A patient was admitted with a nodule located in the right upper lobe and measuring 26.7 mm at its greatest diameter on a chest radiograph and computed tomography scan. **c** A radial probe EBUS placed within the target lesion showed a hypoechoic area with numerous hyperechoic dots. **d** Chest radiographs on day 5 showed an increased pneumonic consolidation (arrow) around the suspected tumor in the right upper lobe

Overall, complications related to EBUS-GS during the learning curve occurred in three patients (1.5%): pneumothorax developed in two patients (1.0%) but resolved spontaneously without the need for chest tube drainage (Fig. 2), and one patient (0.5%) suffered pulmonary infection after the procedure (Fig. 3). Within the total group of study patients, none developed pneumothorax requiring chest tube drainage, severe hemorrhage, air embolism, or respiratory failure. There were no premature terminations of the procedure and none of the patients died due to the procedure.

### Durability of the devices

**Fig. 4:**
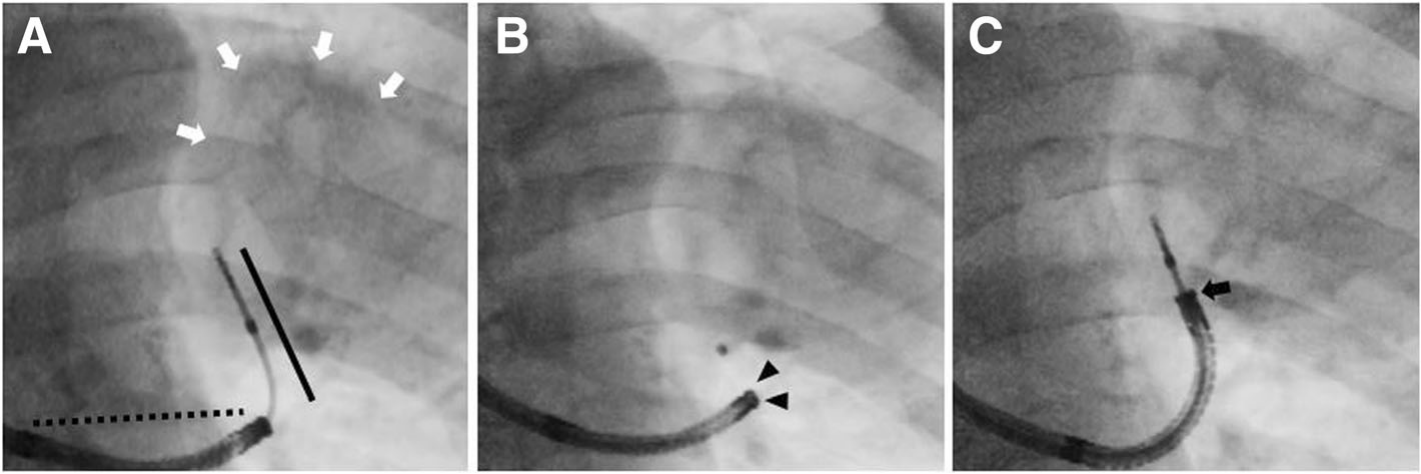
Breakage of the guide sheath (GS). **a** Forceps biopsy via the GS was performed under fluoroscopic guidance after precise identification of the tumor using a radial probe EBUS (white arrow). **b** A kink in the GS (arrowhead) resulting in its dislocation was seen on fluoroscopy. The kink may have been caused by a discordance between the long axes of the bronchoscope (dotted line, **a**) and the GS (black line, **a**). **c** To prevent additional breakage of the GS, a thin bronchoscope was introduced as far as possible close to the target lesion (arrow). Thereafter, the two long axes of the bronchoscope and GS were aligned and the procedure was successfully completed

During the study period, two radial probes EBUS were used by the two physicians and one probe broke. During EBUS-GS, breakage of the GS, observed fluoroscopically, only occurred in one patient (0.5%) (Fig. 4).

## Discussion

This study demonstrated that EBUS-GS is a useful and safe procedure, even when performed by inexperienced physicians. To the best of our knowledge, this is the first report in which the diagnostic yields, learning curve, and safety profile of EBUS-GS during the learning phase were evaluated. We found that EBUS-GS performed by beginners resulted in diagnostic yields comparable to those of experienced physicians [5, 6, 16, 17]. Moreover, the overall complication rate of EBUS-GS in this study was 1.5%, which was not significantly different from the complication rate of 1.3% recorded in a previous study involving 965 peripheral lung lesions [9].

The diagnostic yield of EBUS-GS when performed without any assistance from navigation modalities has been previously reported to be 69.2–77.3% [5, 18]. In this study, the overall diagnostic yield of EBUS-GS performed by beginners was 73.0%. Our results suggest that the accuracy of EBUS-GS does not greatly differ between beginners and experts. In addition, the learning curve analyses showed that the diagnostic yields were stable, even when the procedure was performed by a beginner. Because the diagnostic yields of EBUS-GS are generally a function of the size of the lung lesion [2, 5], we used a CUSUM analysis to assess the two physicians in their diagnostic yields of patients with lung lesions < 30 mm. Our results suggest that EBUS-GS is a stable procedure even when performed by beginners examining small lung lesions.

Interestingly, the graphs of the two physicians crossed the lower decision boundary, indicating that the diagnostic yield improved over time in the analysis of all study subjects (Fig. 1a and b). However, in the CUSUM analysis of the 50 consecutive patients with peripheral lung lesions < 30 mm, the curve of the two physicians remained between the lower and upper decision boundaries (Fig. 1c and d). Therefore, it is expected that the diagnostic yield of EBUS-GS for peripheral lung lesions ≥30 mm improved over time, whereas the diagnostic yield for peripheral lung lesions < 30 mm was stable. From our results, we deduced that larger lesions were associated with early achievement of competence as well as a higher diagnostic yield [3].

A previous meta-analysis of EBUS-GS reported that pooled rates of any pneumothorax or pneumothorax requiring intercostal catheter drainage are 1% and 0.4%, respectively [7]. These low incidences of pneumothorax are an important advantage of EBUS-GS compared to the relatively high incidence of pneumothorax after transthoracic needle biopsy [1, 8, 19]. In our study, the incidence of pneumothorax was 1%, and no patient required the placement of a chest tube for the management of a pneumothorax. These results suggest that even when EBUS-GS is performed by a beginner, the incidence of pneumothorax is much lower than the pneumothorax rate after transthoracic needle aspiration [20]. Pulmonary infection after EBUS-GS is a rare complication, with a risk for 0.5% according to a previous study [9]; the rate was the same in this study. Until now, there has been no clinical guideline or consensus statement regarding prophylactic antibiotics for patients undergoing EBUS-GS. However, the incidence of pulmonary infection in our patients after EBUS-GS was, fortunately low, even when the procedure was performed during the learning phase. In another meta-analysis, respiratory failure after EBUS-GS only occurred in 1 in 2156 patients [6]. In addition, no case of severe hemorrhage or procedure-related deaths have been reported in any of the studies [7, 21, 22]. Likewise, in this study there were no fatal complications, including respiratory failure.

Moreover, we also found that the durability of the radial probe EBUS and GS were tolerable during the learning phase of EBUS-GS. The vulnerability of the radial probe EBUS is well known, and the probe can be used during 50–100 EBUS-GS procedures [18]. In this study, two probes were used by the two physicians, for 100 EBUS-GS procedures each. During that time, one radial probe EBUS broke, but the damage rate was not higher in the EBUS procedures performed by two beginners in this study than that reported elsewhere [18]. In the single case of GS breakage, the two long axes of the bronchoscope and GS were discordant such that the GS bent due to the application of pressure vertical along its long axis (Fig. 4). This situation might have evolved due to the inexperience of the physician. To prevent breakage of the GS, the bronchoscope should be introduced as close as possible to the target lesion.

There were several limitations to our study. First, it was retrospective and conducted at a single center. Although the data were prospectively collected, potential selection bias might have influenced our results. In particular, the proportion of “within the lesion” on the endobronchial ultrasound image and malignant disease in the clinical diagnosis was relatively high in the present study. Previous studies have reported that factors contributing to successful EBUS-GS are “within the lesion” on sonography, a higher proportion of malignant disease in all subjects, and lesion size [5, 21, 23]. We acknowledge possible selective recruitment of patients with a clear bronchus sign on a CT scan; consequently, the proportion of “within” images on endobronchial sonographic images could have increased. The diagnostic yield was well maintained from the beginning of EBUS-GS due to potentially biased selection of patients with the bronchus sign as well as those with malignant disease. Our results suggest that EBUS-GS is a safe, stable, and reproducible procedure, even if performed by beginners, if patient selection is based on the presence of the bronchus sign on a CT scan and a high probability of malignant disease. Second, a navigation system, such as electromagnetic navigation or virtual bronchoscopic navigation, was not used during EBUS-GS. Recent studies have demonstrated that a combined modality made of a navigation system and radial probe EBUS provides a higher diagnostic yield than obtained when each modality is used separately [18, 21]. However, a navigation system is an expensive medical resource and is not available at all of the hospitals. Third, the performance of only two physicians, as beginners in the use of EBUS-GS, was analyzed in this study, which prevents generalization of the results. To verify our findings, a large-scale prospective study of a large-number of beginners of the procedure is needed.

## Conclusions

Recent guidelines recommend the use of radial probe EBUS in patients with peripheral lung nodules [24]. Our results suggest that, unlike many clinical procedures, EBUS-GS, even when performed by an inexperienced physician, is safe with an acceptable diagnostic yield. Moreover, the devices used for EBUS-GS are durable during the learning curve.

## Additional files

Additional file 1: **Figure S1.** Three sets of endobronchial ultrasound images. (DOCX 71 kb)

Additional file 2: **Figure S2.** A representative case. (DOCX 66 kb)

Additional file 3: The definition of cumulative sum analysis. (DOCX 15 kb)

Additional file 4: A detailed description of the cumulative sum analysis. (DOCX 14 kb)

Additional file 5: **Table S1.** Comparison of baseline characteristics between the two study groups. (DOCX 14 kb)

## Abbreviations

CT: Computed tomography
CUSUM: Cumulative sum
EBUS-GS: Radial probe endobronchial ultrasound using a guide sheath
IQR: Interquartile ranges
