# Supplementary material for: Radial probe endobronchial ultrasound using a guide sheath for peripheral lung lesions in beginners
Source: BMC Pulm Med. 2018 Aug 13;18:137. doi: 10.1186/s12890-018-0704-7 (PMC6090614; doi:10.1186/s12890-018-0704-7)
Supplement: Supplementary file 1 — Figure S1. Three sets of endobronchial ultrasound images. (DOCX 71 kb) [file 12890_2018_704_MOESM1_ESM.docx]

Figure S1. Three sets of endobronchial ultrasound images.


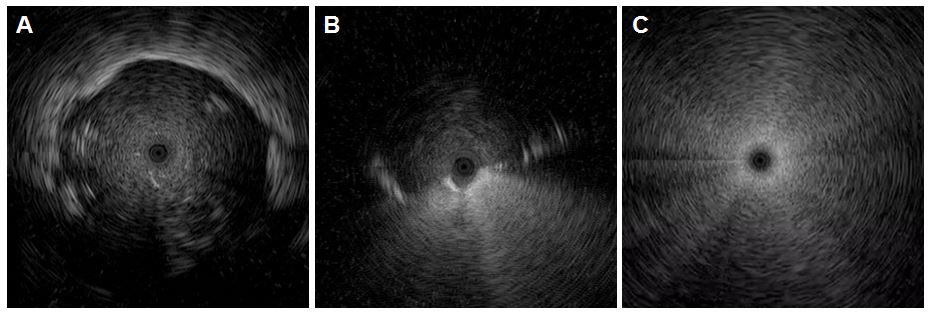
The radial probe endobronchial ultrasound was positioned inside the lesion (A, within), in the bronchus adjacent to the lesion (B, adjacent to), and outside the lesion (C, outside).
