# Supplementary material for: Radial probe endobronchial ultrasound using a guide sheath for peripheral lung lesions in beginners
Source: BMC Pulm Med. 2018 Aug 13;18:137. doi: 10.1186/s12890-018-0704-7 (PMC6090614; doi:10.1186/s12890-018-0704-7)
Supplement: Supplementary file 2 — Figure S2. A representative case. (DOCX 66 kb) [file 12890_2018_704_MOESM2_ESM.docx]

Figure S2. A representative case.

*
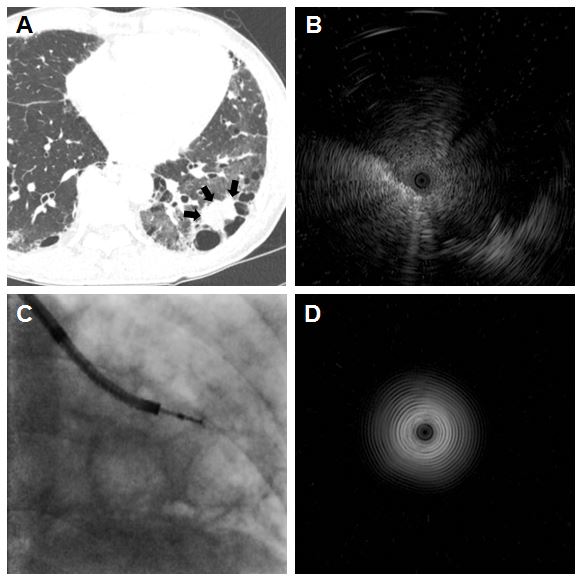
*

A 24-mm lesion suspected of being lung cancer was seen on an axial computed tomography scan in a patient with idiopathic pulmonary fibrosis. Due to lung destruction around the tumor (black arrow) and reduced lung function, it was impossible to perform transthoracic needle biopsy or surgical lung biopsy for histological examination (A). Instead, lung tissue sampling was achieved via peripheral bronchoscopy using radial probe EBUS and a guide sheath. The suspicious lesion was identified on the ultrasound image (B) and transbronchial lung biopsy was performed under X-ray fluoroscopic guidance (C). Small cell lung cancer was diagnosed from the transbronchial lung biopsy obtained using radial probe EBUS. After the procedure, an image of the radial probe EBUS held in the air was taken to ascertain probe breakage (D).

EBUS = endobronchial ultrasound.
