# Supplementary material for: Radial probe endobronchial ultrasound using a guide sheath for peripheral lung lesions in beginners
Source: BMC Pulm Med. 2018 Aug 13;18:137. doi: 10.1186/s12890-018-0704-7 (PMC6090614; doi:10.1186/s12890-018-0704-7)
Supplement: Supplementary file 3 — The definition of cumulative sum analysis. (DOCX 15 kb) [file 12890_2018_704_MOESM3_ESM.docx]

The definition of cumulative sum analysis.

p0 = acceptable failure rate

p1 = unacceptable failure rate

P = ln(p1/p0)

Q = ln((1-p0)/(1-p1))

s = Q/(P+Q)

α = Type 1 failure rate

β = Type 2 failure rate

a = ln((1- β)/α))

b = ln((1-α)/β)

H0 = -b/(P+Q)

H1 = a/(P+Q)
