# Supplementary material for: Radial probe endobronchial ultrasound using a guide sheath for peripheral lung lesions in beginners
Source: BMC Pulm Med. 2018 Aug 13;18:137. doi: 10.1186/s12890-018-0704-7 (PMC6090614; doi:10.1186/s12890-018-0704-7)
Supplement: Supplementary file 4 — A detailed description of the cumulative sum analysis. (DOCX 14 kb) [file 12890_2018_704_MOESM4_ESM.docx]

A detailed description of the cumulative sum analysis.

To determine the reward for a successful procedure (s) and the penalty for a failed procedure (1-s), acceptable (p0) and unacceptable (p1) failure rates were established. Generally, the values of p0 and p1 are determined by the researcher, based on the reference study and clinical decision. In the case of radial probe EBUS-GS, the physicians were considered to have attained procedural competence when the success rate was > 70% (p0 = 0.3). The unacceptable failure rate was set at 40% (p1 = 0.4), in accordance with previous studies.^1,2^ Consequently, the reward and penalty for each procedure were as follows: s = 0.35 (reward for a successful procedure) and 1-s = 0.65 (penalty for a failed procedure). In addition, the values of α (type 1 failure rate) and β (type 2 failure rate) were determined to allow calculation of H0 and H1. Setting type 1 and 2 errors equal to one another is considered the best way to easily interpret CUSUM graphs; therefore, the value of each error in this study was set at 0.1. The results of H0 and H1, which denote lower and upper decision boundaries, were –4.97 and 4.97, respectively. For the further analysis of 50 patients with a peripheral lung lesion < 30 mm in diameter, p0 and p1 were set at 0.33 and 0.43, respectively.^3^ In these cases, the reward and penalty for each procedure were s = 0.38 and 1-s = 0.62. Consequently, H0 and H1 were calculated as –5.15 and 5.15, respectively. The outcome score of each consecutive procedure is presented as *Xn*, and the inherent risk for the procedure as *Xo*. In terms of the results, the CUSUM score is defined by the cumulative sum of *Xn*–*Xo*. The CUSUM graph shows the outcome trend for each physician; an upward projection of the graph denotes a failed performance and a stable or downward projection a stable procedure.

**References**

1. Kurimoto N, Miyazawa T, Okimasa S, Maeda A, Oiwa H, Miyazu Y, Murayama M. Endobronchial ultrasonography using a guide sheath increases the ability to diagnose peripheral pulmonary lesions endoscopically. Chest. 2004;126: 959-65.

2. Eberhardt R, Anantham D, Ernst A, Feller-Kopman D, Herth F. Multimodality bronchoscopic diagnosis of peripheral lung lesions: a randomized controlled trial. *Am* J Respir Crit Care Med. 2007;176: 36-41.

3. Ishida T, Asano F, Yamazaki K, Shinagawa N, Oizumi S, Moriya H, Munakata M, Nishimura M; Virtual Navigation in Japan Trial Group. Virtual bronchoscopic navigation combined with endobronchial ultrasound to diagnose small peripheral pulmonary lesions: a randomised trial. Thorax. 2011; 66: 1072-7.
