# Supplementary material for: Radial probe endobronchial ultrasound using a guide sheath for peripheral lung lesions in beginners
Source: BMC Pulm Med. 2018 Aug 13;18:137. doi: 10.1186/s12890-018-0704-7 (PMC6090614; doi:10.1186/s12890-018-0704-7)
Supplement: Supplementary file 5 — Table S1. Comparison of baseline characteristics between the two study groups. (DOCX 14 kb) [file 12890_2018_704_MOESM5_ESM.docx]

Table S1. Comparison of the baseline characteristics of the two study groups

| Variables | Physician 1  (n = 100) | Physician 2  (n = 100) | *P*-value |
| --- | --- | --- | --- |
| Age, years | 67 (60–73) | 67 (55–74) | 0.683 |
| Male gender | 65 (65) | 64 (64) | 0.883 |
| Mean diameter of lesion, mm | 23 (20–34) | 29 (20–40) | 1.000 |
| Character of lesion on computed tomography |  |  | 0.251 |
| Solid | 81 (81) | 89 (89) |  |
| Part-solid | 16 (16) | 10 (10) |  |
| Ground-glass opacity | 3 (3) | 1 (1) |  |
| Location of the lesion |  |  | 0.089 |
| Right upper lobe | 19 (19) | 35 (35) |  |
| Right middle lobe | 5 (5) | 7 (7) |  |
| Right lower lobe | 26 (26) | 22 (22) |  |
| Left upper division | 23 (23) | 22 (22) |  |
| Left lingular division | 4 (4) | 2 (2) |  |
| Left lower lobe | 23 (23) | 12 (12) |  |
| Endobronchial ultrasound image |  |  | 0.084 |
| Within | 75 (75) | 87 (87) |  |
| Adjacent to | 15 (15) | 9 (9) |  |
| Outside | 10 (10) | 4 (4) |  |
| Number of brush cytology tests | 3 (3–3) | 3 (3–3) | 0.317 |
| Number of forceps biopsies | 6 (6–7) | 6 (6–7) | 0.317 |
| Overall procedural time, min | 21 (15–26) | 19 (13–24) | 0.439 |

Data are medians (interquartile ranges) or numbers (percentages).
